# Apoptosis Disorder, a Key Pathogenesis of HCMV-Related Diseases

**DOI:** 10.3390/ijms22084106

**Published:** 2021-04-15

**Authors:** Zhongjie Yu, Yashuo Wang, Lili Liu, Xianjuan Zhang, Shasha Jiang, Bin Wang

**Affiliations:** 1 Department of Special Medicine, School of Basic Medicine, Qingdao Medical College, Qingdao University, Qingdao 266000, China; zhongjiework@163.com; 2 College of Life Sciences, Qingdao University, Qingdao 266000, China; wangyashuo_fraya@outlook.com; 3 Department of Basic Medicine, School of Basic Medicine, Qingdao Medical College, Qingdao University, Qingdao 266000, China; lililiu2016@126.com; 4 Department of Pathogenic Biology, School of Basic Medicine, Qingdao Medical College, Qingdao University, Qingdao 266000, China; 18354226552@139.com (X.Z.); 15844207055@163.com (S.J.)

**Keywords:** HCMV, apoptosis, molecular mechanism, target therapy

## Abstract

Human cytomegalovirus (HCMV) belongs to the β-herpesvirus family, which is transmitted in almost every part of the world and is carried by more than 90% of the general population. Increasing evidence indicates that HCMV infection triggers numerous diseases by disrupting the normal physiological activity of host cells, particularly apoptosis. Apoptosis disorder plays a key role in the initiation and development of multiple diseases. However, the relationship and molecular mechanism of HCMV-related diseases and apoptosis have not yet been systematically summarized. This review aims to summarize the role of apoptosis in HCMV-related diseases and provide an insight into the molecular mechanism of apoptosis induced by HCMV infection. We summarize the literature on HCMV-related diseases and suggest novel strategies for HCMV treatment by regulating apoptosis.

## 1. Introduction

Human cytomegalovirus (HCMV) is a β-herpesvirus, also known as human herpes virus 5. Compared with other human herpesviruses, the prevalence of HCMV is high, and more than 90% of the general population is an HCMV carrier [1,2]. HCMV infection can disrupt homeostasis by affecting the host cell autophagy, apoptosis [3], proliferation, invasion [4], angiogenesis, and immune response [5]. Increasing evidence indicates that HCMV causes the occurrence and progression of inflammation [6], atherosclerosis [7], Crohn’s disease [8], and various cancers [9]. More seriously, HCMV can trigger life-threatening diseases in immunosuppressed individuals [10]. Thus, it is necessary to elucidate the pathogenic mechanism of HCMV and discover novel targets and strategies for anti-HCMV treatment.

Apoptosis is a form of programmed cell death, which is essential for maintaining normal physiology and tissue function by cleaning up cells that are damaged, dysfunctional, or no longer necessary [11,12]. The survival and accumulation of damaged or unnecessary cells contribute to numerous diseases, such as cancers, in addition to immunological, neurodegenerative, cardiovascular, and infectious diseases [11,13]. Apoptosis generally occurs through two distinct pathways: intrinsic and extrinsic [14,15]. Interestingly, both extrinsic and intrinsic apoptotic pathways can be activated by pathogenic infection [16].

Most reviews of HCMV studies focus on the viral and immune response aspects of pathogenesis and neglect the critical role of HCMV in cellular and molecular events. One of the most important of these events is apoptosis. Multiple products of the HCMV genome can interfere with the survival of infected cells by regulating the onset of apoptosis pathways [17]. Impairing the apoptosis of infected cells is a critical step in HCMV replication, which mediates the occurrence of HCMV-related diseases. Therefore, regulating the apoptosis of infected cells may provide a novel strategy for the treatment of HCMV-related diseases. In this review, we summarize the detailed regulatory role of HCMV in apoptosis and provide novel insight into the effective treatment of HCMV-related diseases.

## 2. The Prevalence and Perniciousness of HCMV

Cytomegalovirus was first isolated by Smith and Rowe in 1956 [18,19] and, in 1990, Chee et al. reported the annotated draft of the HCMV genome [20]. Recent research shows that HCMV consists of double-stranded linear DNA. The HCMV genome is 236 kb, which can encode 167 genes and translate more than 750 open reading frames [21]. Most of the genetic products are strongly correlated to HCMV infection and prevalence. The major transmission routes of HCMV include saliva, semen, urine, placental transfer, breastfeeding, blood transfusion, organ transplantation, and hematopoietic stem cell transplantation [22,23,24]. Because of these numerous routes, HCMV has easily spread globally, and approximately 100% of adults in developing countries are carriers [25,26].

**Figure 1:**
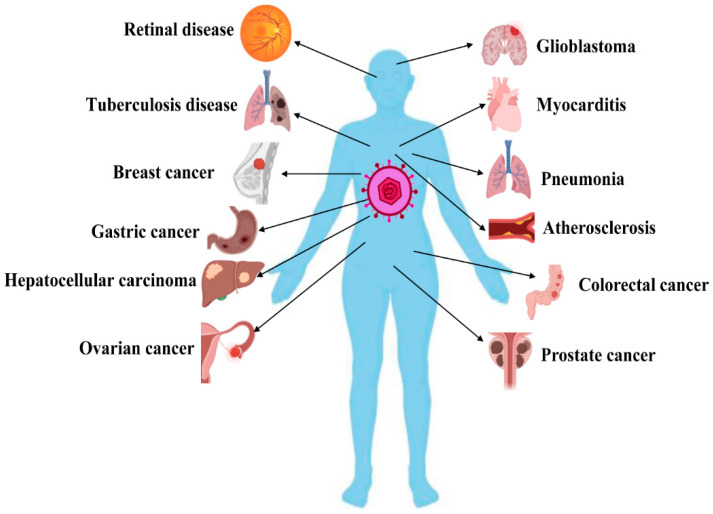
Overview of human cytomegalovirus (HCMV)-related diseases. HCMV infection causes the occurrence and progression of numerous diseases by inducing apoptosis disorder. This figure was created by using Biorender.com (accessed on 5 April 2021).

Clinically, most of those (approximately 90%) who develop a primary HCMV infection are symptomless, and only a few people show symptoms such as asthenia, headache, chills, fever, and sweating [27,28]. However, HCMV can infect most cell types/organs and results in more morbidity and mortality compared to any other herpes virus. HCMV infection is seriously life-threatening to immunocompromised patients, organ transplantation patients, and patients undergoing chemotherapy [29]. Congenital infection often leads to serious complications, including visual disorders, sensorineural deafness, neurodevelopmental impairment, developmental delay, and epilepsy [30,31,32]. Moreover, several studies have shown that HCMV infection causes the occurrence and progression of several chronic diseases, including autoimmune disease (AID) [33], tuberculosis [34], atherosclerosis [7], mental disorders [35], age-related macular degeneration (AMD) [36], pneumonitis, and myocarditis [37]. In particular, many studies have revealed that HCMV infection is related to various cancers, such as cervical cancer [38], breast cancer [39], colorectal cancer [40], ovarian cancer [41], prostate cancer [42], squamous cell carcinoma [43], lymphoma [44], glioblastoma [45], medulloblastoma [46], and neuroblastoma [47], as well as poor outcomes. A list of HCMV-related diseases is provided in Figure 1.

During HCMV infection, a large number of proteins are expressed, which interfere with the normal physiological activity of host cells and ensure HCMV replication [48]. Studies have reported that HCMV infection can significantly affect the expression profile of cytokines [36,49] and disrupt intracellular calcium and adenylate triphosphate homeostasis [50], affecting cell differentiation [51], apoptosis [3], proliferation, and migration [52]. Most importantly, apoptosis disorder induced by HCMV infection is strongly associated with the pathogenesis of numerous diseases.

## 3. HCMV Causes Numerous Diseases by Inducing Apoptosis Disorder

Cell death, or apoptosis, is considered the first line of defense and a key defense mechanism against viral infection due to the fact that it inhibits the spread of infection from infected to uninfected cells. HCMV is a slow-replicating virus, which has evolved and acquired anti-apoptotic genes [17]. These genes encode several apoptosis inhibitors, such as pUL38 and UL138, which allow them to abrogate apoptosis to ensure HCMV replication [53,54]. Apoptosis disorder results in numerous diseases.

### 3.1. Immune System Diseases

AID is a complex disorder of the immune function caused by both genetic and environmental factors. HCMV is a key pathogen and plays a critical role in the onset and progress of AID by regulating the apoptosis of its host cells [55].

#### 3.1.1. Systemic Lupus Erythematosus

Systemic lupus erythematosus (SLE) is a chronic, systemic autoimmune disease, with a prevalence of 0.3 to 23.2 cases per 100,000 [56]. HCMV is one of the environment-related pathogenetic factors of SLE and contributes to the development of SLE. HCMV US31, UL55, and pp65 may play a vital role in the development of SLE by causing abnormal cellular events in its host cells [57,58,59]. One such event is apoptosis, which is considered to be involved in the pathogenesis of SLE. Neo et al. revealed that viral antigen UL44 is redistributed to the cell surface during HCMV-induced apoptosis and then accelerates the development of SLE [60]. These results suggest that apoptosis induced by HCMV infection is significantly associated with the progression of SLE.

#### 3.1.2. Systemic Sclerosis

Systemic sclerosis (SSc) is mainly characterized by skin involvement that often affects multiple organ systems [61]. Among the numerous pathogenetic factors, HCMV is the key factor enhancing the progress of SSc [62]. Evidence shows that HCMV UL83 and UL94 are associated with SSc [63,64]. Arcangeletti et al. indicated that HCMV-specific CD8+ T cells play an important role in the development of SSc [65]. Pastano et al. pointed out that HCMV infection induces both endothelial cell apoptosis and fibroblast proliferation, promoting the progress of SSc [66]. These results show that HCMV infection causes the development of SSc by regulating the apoptosis and proliferation of its host cells.

### 3.2. Pneumonia

Acute interstitial pneumonia (AIP) is an idiopathic pulmonary disease that can cause rapid, progressive dyspnea and respiratory failure. HCMV is a key pathogenetic factor of AIP. Chen et al. reported that HCMV infection disrupts lung fibroblast proliferation and apoptosis by regulating the WNT signaling pathway and leads to AIP [67]. In addition, Maidji et al. revealed that HCMV replication triggers apoptosis and inhibits the production of surfactant proteins in the alveolar epithelium, finally resulting in pneumonia and acute lung injury [68]. These findings highlight the critical role of apoptosis induced by HCMV infection in the occurrence and development of pneumonia.

### 3.3. Atherosclerosis

Atherosclerosis is still one of the main causes of death in both developed and developing countries [69]. One of the main pathogenetic factors of atherosclerosis is apoptosis disorder induced by HCMV infection. Studies have reported that HCMV infection can activate atherosclerosis-relevant factors involved in atherosclerotic plaque rupture and myocardial infarction [70]. Tanaka et al. revealed that HCMV immediately-early 2 (IE2)-84 viral protein abrogates p53-mediated apoptosis and leads to smooth muscle cell accumulation, thereby contributing to restenosis and atherosclerosis [71]. Fan et al. indicated that *HCMV-miR-US25-1* levels are upregulated in its host cells, and that deteriorates oxidized low-density lipoprotein induced the apoptosis of endothelial cells, promoting the development of atherosclerosis [72]. Furthermore, HCMV-derived proteins US28 and UL122 induced endothelial cell damage and apoptosis, which accelerated the process of atherosclerosis [73]. These results imply that HCMV causes the apoptosis of endothelial cells and contributes to the initiation and progression of atherosclerosis.

### 3.4. Cancers

Compared with the diseases discussed above, cancer is more strongly linked to apoptosis disorder induced by HCMV infection. Increasing evidence indicates that the products of the HCMV genome cause the dysregulation of apoptosis and are involved in the oncogenesis [74], for example, glioblastoma [4], breast cancer [75], and leukemia [76].

#### 3.4.1. Glioblastoma

Glioblastoma is the most common and malignant tumor. It occurs primarily in the nervous system and has a high morbidity rate. Studies have reported that HCMV infection contributes to the development of glioblastoma by interfering with apoptosis. Liang et al. revealed that *HCMV-miR-UL112-3p* enhances the proliferation, clone formation, migration, and invasion and suppresses the apoptosis of glioblastoma cells by targeting and downregulating tumor suppressor candidate 3 and then accelerates the progression of glioblastoma [77]. Furthermore, HCMV infection suppressed the apoptosis of glioblastoma cells by increasing the expression level of activating transcription factor 5 (ATF5) and the B cell lymphoma/leukemia-2 (Bcl-2)-to-Bcl-2-associated X (BAX) protein ratio [78]. These results indicate that HCMV infection promotes the development of glioblastoma by suppressing apoptosis.

#### 3.4.2. Neuroblastoma

Neuroblastoma is a pediatric cancer entity strongly associated with HCMV infection. Studies have reported that HCMV infection protects neuroblastoma cells from cytotoxic-agent-induced apoptosis, which might result in the failure of therapy in some neuroblastoma patients [79].

#### 3.4.3. Breast Cancer

Breast cancer is the most common cancer affecting women worldwide [80]. HCMV infection is one of the key factors contributing to the progression of breast cancer. Valle Oseguera et al. reported that cmv interleukin 10 (IL-10) can bind to the IL-10 receptor of breast cancer cells and can then activate the signal transducer and activator of transcription 3 (STAT3), which protect breast cancer cells from etoposide-induced apoptosis and also promote cancer cell proliferation [81].

#### 3.4.4. Acute Myeloid Leukemia

Acute myeloid leukemia is a group of heterogeneous diseases that is only cured in a small number of patients [82]. Unlike its carcinogenesis in other types of cancers, HCMV can significantly suppress the proliferation of acute myeloid leukemia cells, enhance the expression of HLA-class-II-molecules, and increase apoptosis [76].

#### 3.4.5. Hepatocellular Carcinoma

Hepatocellular carcinoma (HCC) accounts for 70–90% of primary liver cancer diagnoses and is one of the most common malignancies worldwide [83]. To date, there is no effective therapy for advanced HCC. Kumar et al. revealed that HCMV could provide antitumoral effects in a murine model of HCC. HCMV infection restricted cellular proliferation and enhanced apoptosis by inhibiting the activation of the STAT3-cyclin D1 signaling pathway [84]. In contrast, Lepiller pointed out that HCMV induces the expression of IL-6 and then activates the IL-6R-Janus kinase (JAK)-STAT3 pathway, which results in the up-regulation of cyclin D1 and survivin. All of these cytokines enhance cancer cell proliferation and colony formation, and finally, accelerate the development of HCC [9].

#### 3.4.6. Gastric Cancer

As one of the most prevalent gastrointestinal diseases, gastric cancer (GC) is the main cause of cancer-related deaths worldwide. Unfortunately, most patients are detected at the advanced stage of the disease and lose any chance of recovery [85,86]. Chen et al. reported that HCMV UL138 can act as a tumor inhibitor in GC. UL138 inhibits GC cell viability and induces apoptosis by interacting with heat shock protein 70 (HSP70), suppressing the progression of GC [53].

Overall, apoptosis disorder is strongly linked to numerous diseases. After infection, the products of the HCMV genome lead to abnormal apoptosis, which contributes to or suppresses the development of diseases. These findings reveal that regulating the apoptosis of host cells may provide a novel therapeutic strategy for HCMV-related diseases.

## 4. Molecular Mechanism of Apoptosis Mediated by HCMV Infection

**Figure 2:**
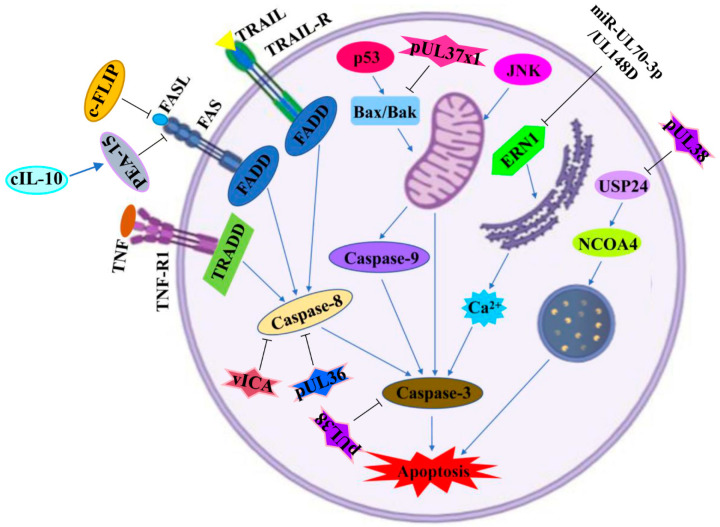
Apoptotic signaling pathways regulated by HCMV infection. A diagram of apoptotic signaling pathways induced (↑) or inhibited (T) by HCMV infection. IE2: immediately-early 2 protein; c-FLIP: cellular FLIP; FASL: Fas cell surface death receptor (FAS) ligand; FADD: FAS-associated death domain; TRAIL: TNF-related apoptosis-inducing ligand; TRAIL-R: TNF-related apoptosis-inducing ligand receptor; TNF: tumor necrosis factor; TNF-R1: tumor necrosis factor receptor 1; TRADD: TNF receptor-1-associated death domain; PEA-15: phosphoprotein enriched in astrocytes-15; cIL-10: cellular interleukin 10; vICA: viral inhibitor of caspase-8 activation; Bax: BCL2-associated X protein 2; Bak: BCL2 homologous antagonist killer; JNK: c-Jun N-terminal kinase; ERN1: endoplasmic-reticulum-to-nucleus signaling 1; USP24: ubiquitin-specific protease 24; NCOA4: nuclear receptor coactivator 4. This figure was created on Biorender.com.

The mechanism underlying the induction of apoptosis by HCMV infection is quite complex. The products of the HCMV genome disrupt the normal physiological activities of its host cells and then result in apoptosis disorder by mediating both extrinsic and intrinsic signaling pathways. The extrinsic pathway mainly involves special death receptors by activating inward signals. Unlike the extrinsic pathway, the intrinsic pathway depends on intracellular organelles, such as the mitochondria and the endoplasmic reticulum (ER) [87,88]. The apoptotic signaling pathways and key effector genes/proteins regulated by HCMV infection are shown in Figure 2 and Table 1.

### 4.1. Extrinsic Pathway

The extrinsic apoptotic pathway mainly includes extracellular ligands and death receptors, such as tumor necrosis factor receptor 1 (TNF-R1), TNF-related apoptosis-inducing ligand receptor (TRAIL-R), and Fas cell surface death receptor (FAS) [103]. Ligands bind with death receptors, which leads to the formation of a death-inducing signaling complex (DISC), and consequently caspase activation, eventually followed by apoptosis [104].

Studies have reported that HCMV infection can evade cell death through the extrinsic apoptotic pathway. FAS played a critical role in the clearance of virus-infected cells by mediating apoptosis. HCMV infection suppressed the cell surface expression of FAS and, consequently, protected infected cells against FAS-mediated apoptosis [105]. Studies have also reported that HCMV inhibits p73-dependent FAS-mediated apoptosis and contributes to the survival of its host cells [106]. Poole et al. demonstrated that cellular IL-10 (cIL-10) is a key survival factor that can upregulate phosphoprotein enriched in astrocytes-15 (PEA-15) and then protect CD34+ progenitor cells from FAS-mediated apoptosis [107].

HCMV protein IE2 activated cellular FLIP (c-FLIP), which suppressed the activation of caspase-8 and caspase-3 by decreasing the FAS ligand (FASL) in HCMV-infected human retinal cells. Meanwhile, IE2 played a key role in resistance to TRAIL-mediated cell death through the phosphatidylinositol 3-kinase (PI3K) pathway [108].

The viral inhibitor of caspase-8 activation (vICA) is conserved in both HCMV and murine cytomegalovirus (MCMV). Chaudhry et al. revealed that vICA prevents death-receptor-induced apoptosis by inhibiting the activation of caspase-8 and pro-apoptotic signaling [89]. Similarly, McCormick et al. indicated that, in response to infection, vICA can inhibit the activation of both caspase-dependent and caspase-independent apoptotic pathways [109]. Moreover, HCMV pUL36 is regarded as a multifunctional inhibitor that can degrade mixed-lineage kinase domain-like protein (MLKL), prevent proteolytic activation of procaspase-8, and then suppress both necroptosis and apoptosis during HCMV infection [90].

In contrast, Chien et al. revealed that MCMV-infected eyes show significant amounts of TNF-α, TNF receptors 1 and 2, active caspase-8 and caspase-3, TRAIL, TRAIL-R, FAS, and FASL. In addition, MCMV-infected eyes also upregulate the expression of receptor-interacting protein (RIP1, RIP3), caspase-1, IL-1β, and IL-18. These results demonstrate that apoptosis, necroptosis, and pyroptosis are activated and participate in the development of MCMV-related retinal disease [110].

### 4.2. Intrinsic Pathway

The intrinsic apoptotic pathway responds to death stimuli, such as DNA damage, chemotherapeutic agents, serum starvation, and viral infection. The intrinsic pathway depends on organelle dysfunction; for instance, ER stress, lysosomal dysfunction, and mitochondrial dysfunction all trigger apoptosis [111].

Studies have reported that HCMV promotes the survival of human embryonic lung fibroblasts by activating the mitogen-activated protein kinase/extracellular-regulated protein kinase (MAPK/ERK) signaling pathway. BCL2-associated athanogene 1 (Bag-1) was upregulated in a MAPK/ERK-dependent fashion and was indispensable suppression of apoptosis in HCMV-infected cells [112]. In contrast, HCMV infection induced human retinal pigment epithelium cell apoptosis by activating caspase-3 and the poly ADP-ribose polymerase (PARP) pathway, which caused severe visual impairment [113]. Dou et al. pointed out that HCMV infection reduces the viability of megakaryocytes by promoting caspase-3-dependent apoptosis via the activation of the c-Jun N-terminal kinase (JNK) signaling pathway [114]. More interestingly, HCMV infection had antitumoral effects by activating the intrinsic apoptotic pathway. HCMV restricted HepG2 cell proliferation decreased colony formation and enhanced intrinsic apoptosis by activating caspase-9 and caspase-3 [84].

#### 4.2.1. Mitochondrial Pathway

The mitochondria play a prominent role in metabolism, as well as being involved in the regulation of apoptosis. Due to different stresses, such as viral infection, apoptosis-related proteins transfer to the mitochondria and further activate and initiate mitochondrial apoptosis. Zhang et al. described a novel anti-apoptotic mechanism of HCMV infection. HCMV pUL37x1 was the potent viral mitochondrion-localized inhibitor of apoptosis (vMIA). vMIA retargeted Bax to the mitochondrion-associated membrane and resulted in increased ubiquitination and proteasome-mediated degradation of Bax, which enhanced the survival of infected cells [91].

#### 4.2.2. Endoplasmic Reticulum Pathway

The endoplasmic reticulum is the center where proteins are modified and folded and where calcium is stored. Endoplasmic reticulum dysfunction promotes the occurrence of apoptosis. During HCMV infection, the ataxia telangiectasia mutant contributed to the activation of p53, and p53 further stimulated Bax and Bak expression, as well as caspase-3 activation, resulting in human aortic endothelial cell (HAEC) dysfunction and apoptosis [115]. Studies have reported that HCMV protein pUL38 prevents cell death by maintaining calcium homeostasis in the endoplasmic reticulum [116]. Moreover, HCMV employs its microRNA against intrinsic apoptosis. Babu et al. indicated that *miR-UL70-3p* and *miR-UL148D* could potentially target the pro-apoptotic gene endoplasmic-reticulum-to-nucleus signaling 1 (ERN1) and then suppress the initiation of endoplasmic-reticulum-stress-induced apoptosis [3].

#### 4.2.3. Lysosome Pathway

Lysosomes play a critical role in multiple cellular events, for example, the degradation of biomacromolecules and the regulation of autophagy. HCMV pUL38 has been shown to prevent cell death via abrogating cellular stress responses. Sun et al. pointed out that during HCMV infection, the ferritinophagy-related protein nuclear receptor coactivator 4 (NCOA4) and lysosomal ferritin degradation are regulated by ubiquitin-specific protease 24 (USP24). pUL38 bound with and antagonized the role of USP24 and then reduced ferritinophagy, finally protecting cells against lysosomal dysfunction-induced cell death [54].

## 5. Novel Treatment Strategies for HCMV-Related Diseases

HCMV infection causes apoptosis disorder, which is involved in the initiation and progression of numerous diseases. Importantly, both positive and negative modulators of apoptosis have attracted researchers’ interest in translating these discoveries from the laboratory to clinical applications in order to improve human health [13]. Based on the data we summarized above, regulation of apoptosis may be a novel strategy for HCMV-related disease treatment.

Leflunomide is an exciting, novel drug for cytomegalovirus infection, which significantly inhibited HCMV-infection-induced apoptosis and played an important role in the treatment of patients with HCMV infection. The study results provided a new way to fight immune dysfunction induced by HCMV infection [117]. In contrast, Biolatti et al. demonstrated that strigolactones exert their antiviral properties by inducing the apoptosis of HCMV-infected cells [118]. Similarly, Mo et al. revealed that treatment with chloroquine remarkably enhances the expression of cleaved caspase-3 in MCMV-infected retinal pigment epithelial cells and contributes to the apoptosis of infected cells [119]. These studies imply that antiviral drugs resist HCMV infection by regulating apoptosis, whether promoting or inhibiting it.

Furthermore, according to the molecular mechanism of apoptosis, multiple vital proteins may be targets for the treatment of HCMV-related diseases. For instance, Zhao et al. indicated that HCMV IE86 promotes the expression of heterogeneous ribonucleoprotein A2/B1 (hnRNP A2/B1) in glioma cells and then prevents apoptosis and contributes to cell proliferation by mediating the alternative splicing of Bcl-x. Knockdown of the expression of hnRNP A2/B1 greatly weakened IE86-mediated apoptosis and cell proliferation [92]. HCMV IE2 promoted the expression of anti-apoptotic genes, such as Mcl-1 and Bcl-2, further suppressed apoptosis, and enhanced the survival and proliferation of smooth muscle cells, which are involved in HCMV-related atherosclerosis [7]. Thus, specifically inhibiting the expression of effector proteins of HCMV may exert positive effects during the treatment of HCMV-related diseases.

HCMV UL138 inhibited GC cell viability and induced apoptosis by reducing the expression level of Bcl-2 protein and promoting the activation of cleaved caspase-9 and caspase-3. In addition, UL138 efficiently prevented GC growth in a xenograft animal model [53]. These findings provided a novel potential therapeutic strategy for GC treatment. More interestingly, Yang et al. pointed out that HCMV glycoprotein B plays no role in cell apoptosis and proliferation but suppresses breast cancer cell migration by downregulating the transforming growth factor (TGF)-β/Smad signaling pathway [75]. Valle Oseguera and Spencer reported that cmvIL-10 promotes the activation of STAT3, which further inhibits apoptosis, increases proliferation, and increases the chemo-resistance of breast cancer cells [81]. These studies revealed the significant role of cytokines in the progression of HCMV-induced breast cancer. In addition, these data may accelerate the development of novel drugs against breast cancer, such as anti-TGF-β and anti-STAT3 agents.

## 6. Conclusions and Future Perspectives

Apoptosis is an essential biological process that not only plays a key role in maintaining natural processes for the growth, development, and health of organisms but also contributes to the initiation and progression of multiple diseases, such as HCMV-related diseases. HCMV is one of the most common pathogens, and it causes numerous diseases by disrupting the apoptosis process through both extrinsic and intrinsic pathways [91,106]. Although the mechanism underlying HCMV-related apoptosis has been well documented, the composition of HCMV is complex, and each component has entirely different effects on apoptosis. Hitherto, the mechanisms of these effects were not completely revealed and should be explored further. As reviewed here, recent evidence has revealed much about HCMV’s impact on disease progression through apoptosis. Understanding the mechanisms of apoptosis and the crosstalk between apoptosis and diseases induced by HCMV infection may help develop innovative therapeutic strategies that will contribute to improving the treatment of patients with HCMV infection. Therefore, it is essential to keep investigating and elucidating the role of apoptosis in HCMV-related diseases.

## Figures and Tables

**Table 1:** The summary of effector gene/proteins regulated by HCMV.

No.	Virus	Key Gene/Protein	Expression Phase	Expression Levels	Host Tissue/Cell Line	Effector Gene/Protein	Results (Impact Apoptosis)	Reference
1	HCMV	*miR-UL70-3p*/ *miR-UL148D*	Latent phases	↑	/	MOAP1/PHAP/ERN1	↓	[3]
2	HCMV	IE2	Immediate early phases	↑	Rat aortic smooth muscle cell	Mcl-1/Bcl-2	↓	[7]
3	HCMV	UL36	Immediate early phases	↑	THP-1 cells	Caspase-8	↓	[17]
4	HCMV	UL138	Latent phases	↓	Gastric cancer cell	HSP70	↑	[53]
5	HCMV	pUL38	Immediate early phases	↑	Human embryonic lung fibroblasts	USP24/NCOA4	↓	[54]
6	HCMV	* miR-US25-1 *	Late phase	↑	Endothelial cells	BRCC 3	↑	[72]
7	HCMV	* miR-UL112-3p *	Immediate early phases	↑	Glioblastoma cell	TUSC3	↓	[77]
8	HCMV	cmvIL-10	Productive and latent phases	↑	Breast cancer cell	Stat3	↓	[81]
9	HCMV/MCMV	vICA	Immediate early phases	↑	CD8 T cell	Caspase-8	↓	[89]
10	HCMV	pUL36	Immediate early phases	↑	Mouse embryonic fibroblasts/ primary human fetal foreskin fibroblasts	MLKL	↓	[90]
11	HCMV	pUL37x1/vMIA	Immediate early phases	↑	Human fibroblasts	Bax	↓	[91]
12	HCMV	IE86	Immediate early phases	↑	U251 cell	hnRNP A2/B1	↓	[92]
13	HCMV	pUS21	Late phase	↑	Human foreskin fibroblasts	Caspase-7/3	↓	[93]
14	HCMV	HCMVAIS	/	↑	Human embryo lung fibroblasts	NOX-2/PARP-1	↑	[94]
15	HCMV	* hcmv-miR-US4-5p *	Immediate early phases	↑	Human embryonic kidney cell/ human embryonic lung fibroblast cell/ human monocytic cell	PAK2	↑	[95]
16	HCMV	IE86	Immediate early phases	↑	Glioma cell	ATF5	↓	[96]
17	HCMV	* hcmv-miR-US4-1 *	/	↑	Human embryonic lung fibroblast	QARS	↑	[97]
18	HCMV	Glycoprotein gB	Immediate early phases	↑	Human peripheral blood monocytes	Akt	↓	[98]
19	HCMV	* hcmv-miR-UL36-5p *	Immediate early phases	↑	Human embryonic kidney cell/human embryonic lung fibroblasts/glioma cell	ANT3	↓	[99]
20	HCMV	* US27 *	Late phase	↑	Human embryonic kidney cell	Bcl-x/AP-1	↓	[100]
21	HCMV	UL141	/	↑	Human fibroblasts/ normal human dermal fibroblasts	TRAIL-R	↓	[101]
22	HCMV	* hcmv-mir-UL148D *	Immediate early phases	↑	Human embryonic kidney cell	IEX-1	↓	[102]

↑: Upregulation; ↓: Downregulation; /: Unknown.

## Data Availability

All data generated or analyzed during this study are included in this published article.

## Abbreviations

HCMV	human cytomegalovirus
MCMV	murine CMV
AID	autoimmune disease
SLE	systemic lupus erythematosus
SSc	systemic sclerosis
AIP	acute interstitial pneumonia
IE2	immediately-early 2
STAT3	signal transducer and activator of transcription 3
hnRNP A2/B1	heterogeneous ribonucleoprotein A2/B1
c-FLIP	cellular FLIP
FAS	Fas cell surface death receptor
FASL	FAS ligand
FADD	FAS-associated death domain
TRAIL	TNF-related apoptosis-inducing ligand
TRAIL-R	TNF-related apoptosis-inducing ligand receptor
TNF	tumor necrosis factor
TNF-R1	tumor necrosis factor receptor 1
TRADD	TNF receptor-1-associated death domain
PEA-15	phosphoprotein enriched in astrocytes-15
cIL-10	cellular interleukin 10
vICA	viral inhibitor of caspase-8 activation
Bax	BCL2-associated X protein 2
Bak	BCL2 homologous antagonist killer
JNK	c-Jun N-terminal kinase
ERN1	reticulum-to-nucleus signaling 1
USP24	ubiquitin-specific protease 24
NCOA4	nuclear receptor coactivator 4
JAK	Janus kinase
MAPK/ERK	mitogen-activated protein kinase/extracellular-regulated protein kinase
Bag-1	BCL2-associated athanogene 1
PARP	poly ADP-ribose polymerase
TGF	transforming growth factor

## References

[1] Clement M., Humphreys I.R. (2019). Cytokine-Mediated Induction and Regulation of Tissue Damage During Cytomegalovirus Infection. Front Immunol..

[2] Geisler J., Touma J., Rahbar A., Söderberg-Nauclér C., Vetvik K. (2019). A Review of the Potential Role of Human Cytomegalovirus (HCMV) Infections in Breast Cancer Carcinogenesis and Abnormal Immunity. Cancers.

[3] Babu S.G., Pandeya A., Verma N., Shukla N., Kumar R.V., Saxena S. (2014). Role of HCMV *miR-UL70-3p* and *miR-UL148D* in overcoming the cellular apoptosis. Mol. Cell. Biochem..

[4] Song J., Ma Q., Hu M., Qian D., Wang B., He N. (2018). The Inhibition of *miR-144-3p* on Cell Proliferation and Metastasis by Targeting TOP2A in HCMV-Positive Glioblastoma Cells. Molecules.

[5] Joseph G.P., McDermott R., Baryshnikova M.A., Cobbs C.S., Ulasov I.V. (2017). Cytomegalovirus as an oncomodulatory agent in the progression of glioma. Cancer Lett..

[6] Landolfo S., Gariglio M., Gribaudo G., Lembo D. (2003). The human cytomegalovirus. Pharmacol. Ther..

[7] Liao X.H., Dong X., Wu C., Wang T., Liu F., Zhou J., Zhang T.C. (2014). Human cytomegalovirus immediate early protein 2 enhances myocardin-mediated survival of rat aortic smooth muscle cells. Virus Res..

[8] Kapoor A., Forman M., Arav-Boger R. (2014). Activation of nucleotide oligomerization domain 2 (NOD2) by human cytomegalovirus initiates innate immune responses and restricts virus replication. PLoS ONE.

[9] Lepiller Q., Abbas W., Kumar A., Tripathy M.K., Herbein G. (2013). HCMV activates the IL-6-JAK-STAT3 axis in HepG2 cells and primary human hepatocytes. PLoS ONE.

[10] de Jong M.D., Galasso G.J., Gazzard B., Griffiths P.D., Jabs D.A., Kern E.R., Spector S.A. (1998). Summary of the II International Symposium on Cytomegalovirus. Antivir. Res..

[11] Lu T., Aron L., Zullo J., Pan Y., Kim H., Chen Y., Yang T.H., Kim H.M., Drake D., Liu X.S. (2014). REST and stress resistance in ageing and Alzheimer’s disease. Nature.

[12] Elmore S. (2007). Apoptosis: A review of programmed cell death. Toxicol. Pathol..

[13] Singh R., Letai A., Sarosiek K. (2019). Regulation of apoptosis in health and disease: The balancing act of BCL-2 family proteins. Nat. Rev. Mol. Cell Biol..

[14] Galluzzi L., Vitale I., Aaronson S.A., Abrams J.M., Adam D., Agostinis P., Alnemri E.S., Altucci L., Amelio I., Andrews D.W. (2018). Molecular mechanisms of cell death: Recommendations of the Nomenclature Committee on Cell Death 2018. Cell Death Differ..

[15] Roy S., Nicholson D.W. (2000). Cross-talk in cell death signaling. J. Exp. Med..

[16] Salaun B., Romero P., Lebecque S. (2007). Toll-like receptors’ two-edged sword: When immunity meets apoptosis. Eur. J. Immunol..

[17] Chaudhry M.Z., Kasmapour B., Plaza-Sirvent C., Bajagic M., Casalegno Garduño R., Borkner L., Lenac Roviš T., Scrima A., Jonjic S., Schmitz I. (2017). UL36 Rescues Apoptosis Inhibition and In vivo Replication of a Chimeric MCMV Lacking the *M36* Gene. Front. Cell. Infect. Microbiol..

[18] Smith M.G. (1956). Propagation in tissue cultures of a cytopathogenic virus from human salivary gland virus (SGV) disease. Proc. Soc. Exp. Biol. Med. Soc. Exp. Biol. Med..

[19] Rowe W.P., Hartley J.W., Waterman S., Turner H.C., Huebner R.J. (1956). Cytopathogenic agent resembling human salivary gland virus recovered from tissue cultures of human adenoids. Proc. Soc. Exp. Biol. Med. Soc. Exp. Biol. Med..

[20] Chee M.S., Bankier A.T., Beck S., Bohni R., Brown C.M., Cerny R., Horsnell T., Hutchison C.A., Kouzarides T., Martignetti J.A. (1990). Analysis of the protein-coding content of the sequence of human cytomegalovirus strain AD169. Curr. Top. Microbiol. Immunol..

[21] Stern-Ginossar N., Weisburd B., Michalski A., Le V.T., Hein M.Y., Huang S.X., Ma M., Shen B., Qian S.B., Hengel H. (2012). Decoding human cytomegalovirus. Science.

[22] Cannon M.J., Schmid D.S., Hyde T.B. (2010). Review of cytomegalovirus seroprevalence and demographic characteristics associated with infection. Rev. Med. Virol..

[23] Pass R.F. (1985). Epidemiology and transmission of cytomegalovirus. J. Infect. Dis..

[24] Ho M. (1990). Epidemiology of cytomegalovirus infections. Rev. Infect. Dis..

[25] Griffiths P., Baraniak I., Reeves M. (2015). The pathogenesis of human cytomegalovirus. J. Pathol..

[26] Staras S.A., Dollard S.C., Radford K.W., Flanders W.D., Pass R.F., Cannon M.J. (2006). Seroprevalence of cytomegalovirus infection in the United States, 1988-1994. Clin. Infect. Dis. Off. Publ. Infect. Dis. Soc. Am..

[27] Wreghitt T.G., Teare E.L., Sule O., Devi R., Rice P. (2003). Cytomegalovirus infection in immunocompetent patients. Clin. Infect. Dis. Off. Publ. Infect. Dis. Soc. Am..

[28] Abgueguen P., Delbos V., Ducancelle A., Jomaa S., Fanello S., Pichard E. (2010). Venous thrombosis in immunocompetent patients with acute cytomegalovirus infection: A complication that may be underestimated. Clin. Microbiol. Infect..

[29] Andrei G., De Clercq E., Snoeck R. (2008). Novel inhibitors of human CMV. Curr. Opin. Investig. Drugs.

[30] Britt W. (2008). Manifestations of human cytomegalovirus infection: Proposed mechanisms of acute and chronic disease. Curr. Top. Microbiol. Immunol..

[31] Boppana S.B., Fowler K.B., Pass R.F., Rivera L.B., Bradford R.D., Lakeman F.D., Britt W.J. (2005). Congenital cytomegalovirus infection: Association between virus burden in infancy and hearing loss. J. Pediatrics.

[32] Burny W., Liesnard C., Donner C., Marchant A. (2004). Epidemiology, pathogenesis and prevention of congenital cytomegalovirus infection. Expert Rev. Anti-Infect. Ther..

[33] Rider J.R., Ollier W.E., Lock R.J., Brookes S.T., Pamphilon D.H. (1997). Human cytomegalovirus infection and systemic lupus erythematosus. Clin. Exp. Rheumatol..

[34] Stockdale L., Nash S., Farmer R., Raynes J., Mallikaarjun S., Newton R., Fletcher H.A. (2020). Cytomegalovirus Antibody Responses Associated With Increased Risk of Tuberculosis Disease in Ugandan Adults. J. Infect. Dis..

[35] Lucchese G., Flöel A., Stahl B. (2020). A Peptide Link Between Human Cytomegalovirus Infection, Neuronal Migration, and Psychosis. Front. Psychiatry.

[36] Xu J., Liu X., Zhang X., Marshall B., Dong Z., Liu Y., Espinosa-Heidmann D.G., Zhang M. (2020). Ocular cytomegalovirus latency exacerbates the development of choroidal neovascularization. J. Pathol..

[37] Fitzgerald N.A., Papadimitriou J.M., Shellam G.R. (1990). Cytomegalovirus-induced pneumonitis and myocarditis in newborn mice. A model for perinatal human cytomegalovirus infection. Arch. Virol..

[38] Marinho-Dias J., Sousa H. (2013). Cytomegalovirus infection and cervical cancer: From past doubts to present questions. Acta Med. Port..

[39] Richardson A.K., Walker L.C., Cox B., Rollag H., Robinson B.A., Morrin H., Pearson J.F., Potter J.D., Paterson M., Surcel H.M. (2020). Breast cancer and cytomegalovirus. Clin. Transl. Oncol..

[40] Bai B., Wang X., Chen E., Zhu H. (2016). Human cytomegalovirus infection and colorectal cancer risk: A meta-analysis. Oncotarget.

[41] Shanmughapriya S., Senthilkumar G., Vinodhini K., Das B.C., Vasanthi N., Natarajaseenivasan K. (2012). Viral and bacterial aetiologies of epithelial ovarian cancer. Eur. J. Clin. Microbiol. Infect. Dis..

[42] Samanta M., Harkins L., Klemm K., Britt W.J., Cobbs C.S. (2003). High prevalence of human cytomegalovirus in prostatic intraepithelial neoplasia and prostatic carcinoma. J. Urol..

[43] Saravani S., Kadeh H., Miri-Moghaddam E., Zekri A., Sanadgol N., Gholami A. (2015). Human Cytomegalovirus in Oral Squamous Cell Carcinoma in Southeast of Iran. Jundishapur J. Microbiol..

[44] Mehravaran H., Makvandi M., Samarbaf Zade A., Neisi N., Kiani H., Radmehr H., Shahani T., Hoseini S.Z., Ranjbari N., Nahid Samiei R. (2017). Association of Human Cytomegalovirus with Hodgkin’s Disease and Non-Hodgkin’s lymphomas. Asian Pac. J. Cancer Prev..

[45] Lisyany N.I., Klyuchnikova A.A., Belskaya L.N., Lisyany A.A., Gnedkova I.A. (2019). Cytomegaloviruses and malignant brain tumors. Exp. Oncol..

[46] Baryawno N., Rahbar A., Wolmer-Solberg N., Taher C., Odeberg J., Darabi A., Khan Z., Sveinbjörnsson B., FuskevÅg O.M., Segerström L. (2011). Detection of human cytomegalovirus in medulloblastomas reveals a potential therapeutic target. J. Clin. Investig..

[47] Maple P.A.C. (2020). Cytomegalovirus and Epstein-Barr Virus Associations with Neurological Diseases and the Need for Vaccine Development. Vaccines.

[48] Dupont L., Reeves M.B. (2016). Cytomegalovirus latency and reactivation: Recent insights into an age old problem. Rev. Med. Virol..

[49] Rabe T., Lazar K., Cambronero C., Goelz R., Hamprecht K. (2020). Human Cytomegalovirus (HCMV) Reactivation in the Mammary Gland Induces a Proinflammatory Cytokine Shift in Breast Milk. Microorganisms.

[50] Sison S.L., O’Brien B.S., Johnson A.J., Seminary E.R., Terhune S.S., Ebert A.D. (2019). Human Cytomegalovirus Disruption of Calcium Signaling in Neural Progenitor Cells and Organoids. J. Virol..

[51] López-Botet M., Muntasell A., Vilches C. (2014). The CD94/NKG2C+ NK-cell subset on the edge of innate and adaptive immunity to human cytomegalovirus infection. Semin. Immunol..

[52] Liu X., Lin K., Huang X., Xie W., Xiang D., Ding N., Hu C., Shen X., Xue X., Huang Y. (2020). Overexpression of the human cytomegalovirus UL111A is correlated with favorable survival of patients with gastric cancer and changes T-cell infiltration and suppresses carcinogenesis. J. Cancer Res. Clin. Oncol..

[53] Chen W., Lin K., Zhang L., Guo G., Sun X., Chen J., Ye L., Ye S., Mao C., Xu J. (2016). The cytomegalovirus protein UL138 induces apoptosis of gastric cancer cells by binding to heat shock protein 70. Oncotarget.

[54] Sun Y., Bao Q., Xuan B., Xu W., Pan D., Li Q., Qian Z. (2018). Human Cytomegalovirus Protein pUL38 Prevents Premature Cell Death by Binding to Ubiquitin-Specific Protease 24 and Regulating Iron Metabolism. J. Virol..

[55] Halenius A., Hengel H. (2014). Human cytomegalovirus and autoimmune disease. BioMed Res. Int..

[56] Rees F., Doherty M., Grainge M.J., Lanyon P., Zhang W. (2017). The worldwide incidence and prevalence of systemic lupus erythematosus: A systematic review of epidemiological studies. Rheumatology.

[57] Guo G., Ye S., Xie S., Ye L., Lin C., Yang M., Shi X., Wang F., Li B., Li M. (2018). The cytomegalovirus protein US31 induces inflammation through mono-macrophages in systemic lupus erythematosus by promoting NF-κB2 activation. Cell Death Dis..

[58] Chen J., Zhang H., Chen P., Lin Q., Zhu X., Zhang L., Xue X. (2015). Correlation between systemic lupus erythematosus and cytomegalovirus infection detected by different methods. Clin. Rheumatol..

[59] Hsieh A.H., Kuo C.F., Chou I.J., Tseng W.Y., Chen Y.F., Yu K.H., Luo S.F. (2020). Human cytomegalovirus pp65 peptide-induced autoantibodies cross-reacts with TAF9 protein and induces lupus-like autoimmunity in BALB/c mice. Sci. Rep..

[60] Neo J.Y.J., Wee S.Y.K., Bonne I., Tay S.H., Raida M., Jovanovic V., Fairhurst A.M., Lu J., Hanson B.J., MacAry P.A. (2019). Characterisation of a human antibody that potentially links cytomegalovirus infection with systemic lupus erythematosus. Sci. Rep..

[61] Attanasio U., Cuomo A., Pirozzi F., Loffredo S., Abete P., Petretta M., Marone G., Bonaduce D., De Paulis A., Rossi F.W. (2020). Pulmonary Hypertension Phenotypes in Systemic Sclerosis: The Right Diagnosis for the Right Treatment. Int. J. Mol. Sci..

[62] Efthymiou G., Dardiotis E., Liaskos C., Marou E., Scheper T., Meyer W., Daponte A., Daoussis D., Hadjigeorgiou G., Bogdanos D.P. (2019). A comprehensive analysis of antigen-specific antibody responses against human cytomegalovirus in patients with systemic sclerosis. Clin. Immunol..

[63] Marou E., Liaskos C., Efthymiou G., Dardiotis E., Daponte A., Scheper T., Meyer W., Hadjigeorgiou G., Bogdanos D.P., Sakkas L.I. (2017). Increased immunoreactivity against human cytomegalovirus UL83 in systemic sclerosis. Clin. Exp. Rheumatol..

[64] Namboodiri A.M., Rocca K.M., Pandey J.P. (2004). IgG antibodies to human cytomegalovirus late protein UL94 in patients with systemic sclerosis. Autoimmunity.

[65] Arcangeletti M.C., Maccari C., Vescovini R., Volpi R., Giuggioli D., Sighinolfi G., De Conto F., Chezzi C., Calderaro A., Ferri C. (2018). A Paradigmatic Interplay between Human Cytomegalovirus and Host Immune System: Possible Involvement of Viral Antigen-Driven CD8+ T Cell Responses in Systemic Sclerosis. Viruses.

[66] Pastano R., Dell’Agnola C., Bason C., Gigli F., Rabascio C., Puccetti A., Tinazzi E., Cetto G., Peccatori F., Martinelli G. (2012). Antibodies against human cytomegalovirus late protein UL94 in the pathogenesis of scleroderma-like skin lesions in chronic graft-versus-host disease. Int. Immunol..

[67] Chen L., Tang R.Z., Ruan J., Zhu X.B., Yang Y. (2019). Up-regulation of THY1 attenuates interstitial pulmonary fibrosis and promotes lung fibroblast apoptosis during acute interstitial pneumonia by blockade of the WNT signaling pathway. Cell Cycle.

[68] Maidji E., Kosikova G., Joshi P., Stoddart C.A. (2012). Impaired surfactant production by alveolar epithelial cells in a SCID-hu lung mouse model of congenital human cytomegalovirus infection. J. Virol..

[69] Lusis A.J. (2000). Atherosclerosis. Nature.

[70] Prochnau D., Lehmann M., Straube E., Figulla H.R., Rödel J. (2011). Human cytomegalovirus induces MMP-1 and MMP-3 expression in aortic smooth muscle cells. Acta Microbiol. Immunol. Hung..

[71] Tanaka K., Zou J.P., Takeda K., Ferrans V.J., Sandford G.R., Johnson T.M., Finkel T., Epstein S.E. (1999). Effects of human cytomegalovirus immediate-early proteins on p53-mediated apoptosis in coronary artery smooth muscle cells. Circulation.

[72] Fan J., Zhang W., Liu Q. (2014). Human cytomegalovirus-encoded *miR-US25-1* aggravates the oxidised low density lipoprotein-induced apoptosis of endothelial cells. BioMed Res. Int..

[73] Lunardi C., Dolcino M., Peterlana D., Bason C., Navone R., Tamassia N., Tinazzi E., Beri R., Corrocher R., Puccetti A. (2007). Endothelial cells’ activation and apoptosis induced by a subset of antibodies against human cytomegalovirus: Relevance to the pathogenesis of atherosclerosis. PLoS ONE.

[74] Priel E., Wohl A., Teperberg M., Nass D., Cohen Z.R. (2015). Human cytomegalovirus viral load in tumor and peripheral blood samples of patients with malignant gliomas. J. Clin. Neurosci..

[75] Yang R., Liang J., Xu G.X., Ding L.M., Huang H.M., Su Q.Z., Yan J., Li Y.C. (2018). Human cytomegalovirus glycoprotein B inhibits migration of breast cancer MDA-MB-231 cells and impairs TGF-β/Smad2/3 expression. Oncol. Lett..

[76] Koldehoff M., Lindemann M., Ross S.R., Elmaagacli A.H. (2018). Cytomegalovirus induces HLA-class-II-restricted alloreactivity in an acute myeloid leukemia cell line. PLoS ONE.

[77] Liang Q., Wang K., Wang B., Cai Q. (2017). HCMV-encoded *miR-UL112-3p* promotes glioblastoma progression via tumour suppressor candidate 3. Sci. Rep..

[78] Wang T., Qian D., Hu M., Li L., Zhang L., Chen H., Yang R., Wang B. (2014). Human cytomegalovirus inhibits apoptosis by regulating the activating transcription factor 5 signaling pathway in human malignant glioma cells. Oncol. Lett..

[79] Cinatl J., Cinatl J., Vogel J.U., Kotchetkov R., Driever P.H., Kabickova H., Kornhuber B., Schwabe D., Doerr H.W. (1998). Persistent human cytomegalovirus infection induces drug resistance and alteration of programmed cell death in human neuroblastoma cells. Cancer Res..

[80] Jemal A., Bray F., Center M.M., Ferlay J., Ward E., Forman D. (2011). Global cancer statistics. CA Cancer J. Clin..

[81] Valle Oseguera C.A., Spencer J.V. (2014). cmvIL-10 stimulates the invasive potential of MDA-MB-231 breast cancer cells. PLoS ONE.

[82] Döhner H., Weisdorf D.J., Bloomfield C.D. (2015). Acute Myeloid Leukemia. N. Engl. J. Med..

[83] Siegel R.L., Miller K.D., Jemal A. (2019). Cancer statistics, 2019. CA Cancer J. Clin..

[84] Kumar A., Coquard L., Pasquereau S., Russo L., Valmary-Degano S., Borg C., Pothier P., Herbein G. (2016). Tumor control by human cytomegalovirus in a murine model of hepatocellular carcinoma. Mol. Ther. Oncolytics.

[85] Ferlay J., Soerjomataram I., Dikshit R., Eser S., Mathers C., Rebelo M., Parkin D.M., Forman D., Bray F. (2015). Cancer incidence and mortality worldwide: Sources, methods and major patterns in GLOBOCAN 2012. Int. J. Cancer.

[86] Khanipouyani F., Akrami H., Fattahi M.R. (2020). Circular RNAs as important players in human gastric cancer. Clin. Transl. Oncol..

[87] Hu S.J., Jiang S.S., Zhang J., Luo D., Yu B., Yang L.Y., Zhong H.H., Yang M.W., Liu L.Y., Hong F.F. (2019). Effects of apoptosis on liver aging. World J. Clin. Cases.

[88] Zhong H.H., Hu S.J., Yu B., Jiang S.S., Zhang J., Luo D., Yang M.W., Su W.Y., Shao Y.L., Deng H.L. (2017). Apoptosis in the aging liver. Oncotarget.

[89] Chaudhry M.Z., Casalegno-Garduno R., Sitnik K.M., Kasmapour B., Pulm A.K., Brizic I., Eiz-Vesper B., Moosmann A., Jonjic S., Mocarski E.S. (2020). Cytomegalovirus inhibition of extrinsic apoptosis determines fitness and resistance to cytotoxic CD8 T cells. Proc. Natl. Acad. Sci. USA.

[90] Fletcher-Etherington A., Nobre L., Nightingale K., Antrobus R., Nichols J., Davison A.J., Stanton R.J., Weekes M.P. (2020). Human cytomegalovirus protein pUL36: A dual cell death pathway inhibitor. Proc. Natl. Acad. Sci. USA.

[91] Zhang A., Hildreth R.L., Colberg-Poley A.M. (2013). Human cytomegalovirus inhibits apoptosis by proteasome-mediated degradation of Bax at endoplasmic reticulum-mitochondrion contacts. J. Virol..

[92] Zhao R., Hu M., Liang S., Wang B., Yu B., Yang G., Qian D. (2019). IE86 Inhibits the apoptosis and promotes the cell proliferation of glioma cells via the hnRNP A2/B1-mediated alternative splicing of Bcl-x. Int. J. Clin. Exp. Pathol..

[93] Luganini A., Di Nardo G., Munaron L., Gilardi G., Fiorio Pla A., Gribaudo G. (2018). Human cytomegalovirus US21 protein is a viroporin that modulates calcium homeostasis and protects cells against apoptosis. Proc. Natl. Acad. Sci. USA.

[94] Kim J.H., Kim J., Roh J., Park C.S., Seoh J.Y., Hwang E.S. (2018). Reactive oxygen species-induced parthanatos of immunocytes by human cytomegalovirus-associated substance. Microbiol. Immunol..

[95] Shao Y., Qi Y., Huang Y., Liu Z., Ma Y., Guo X., Jiang S., Sun Z., Ruan Q. (2017). Human cytomegalovirus *miR-US4-5p* promotes apoptosis via downregulation of p21-activated kinase 2 in cultured cells. Mol. Med. Rep..

[96] Hu M., Wang B., Qian D., Wang M., Huang R., Wei L., Li L., Zhang L., Liu D.X. (2017). Human cytomegalovirus immediate-early protein promotes survival of glioma cells through interacting and acetylating ATF5. Oncotarget.

[97] Shao Y., Qi Y., Huang Y., Liu Z., Ma Y., Guo X., Jiang S., Sun Z., Ruan Q. (2016). Human cytomegalovirus-encoded *miR-US4-1* promotes cell apoptosis and benefits discharge of infectious virus particles by targeting QARS. J. Biosci..

[98] Cojohari O., Peppenelli M.A., Chan G.C. (2016). Human Cytomegalovirus Induces an Atypical Activation of Akt To Stimulate the Survival of Short-Lived Monocytes. J. Virol..

[99] Guo X., Huang Y., Qi Y., Liu Z., Ma Y., Shao Y., Jiang S., Sun Z., Ruan Q. (2015). Human cytomegalovirus *miR-UL36-5p* inhibits apoptosis via downregulation of adenine nucleotide translocator 3 in cultured cells. Arch. Virol..

[100] Lares A.P., Tu C.C., Spencer J.V. (2013). The human cytomegalovirus *US27* gene product enhances cell proliferation and alters cellular gene expression. Virus Res..

[101] Smith W., Tomasec P., Aicheler R., Loewendorf A., Nemčovičová I., Wang E.C., Stanton R.J., Macauley M., Norris P., Willen L. (2013). Human cytomegalovirus glycoprotein UL141 targets the TRAIL death receptors to thwart host innate antiviral defenses. Cell Host. Microbe.

[102] Wang Y.P., Qi Y., Huang Y.J., Qi M.L., Ma Y.P., He R., Ji Y.H., Sun Z.R., Ruan Q. (2013). Identification of immediate early gene X-1 as a cellular target gene of hcmv-mir-UL148D. Int. J. Mol. Med..

[103] Yin X.M., Ding W.X. (2003). Death receptor activation-induced hepatocyte apoptosis and liver injury. Curr. Mol. Med..

[104] Raducka-Jaszul O., Bogusławska D.M., Jędruchniewicz N., Sikorski A.F. (2020). Role of Extrinsic Apoptotic Signaling Pathway during Definitive Erythropoiesis in Normal Patients and in Patients with β-Thalassemia. Int. J. Mol. Sci..

[105] Seirafian S., Prod’homme V., Sugrue D., Davies J., Fielding C., Tomasec P., Wilkinson G.W.G. (2014). Human cytomegalovirus suppresses Fas expression and function. J. Gen. Virol..

[106] Terrasson J., Allart S., Martin H., Lulé J., Haddada H., Caput D., Davrinche C. (2005). p73-dependent apoptosis through death receptor: Impairment by human cytomegalovirus infection. Cancer Res..

[107] Poole E., Lau J.C.H., Sinclair J. (2015). Latent infection of myeloid progenitors by human cytomegalovirus protects cells from FAS-mediated apoptosis through the cellular IL-10/PEA-15 pathway. J. Gen. Virol..

[108] Chiou S.H., Yang Y.P., Lin J.C., Hsu C.H., Jhang H.C., Yang Y.T., Lee C.H., Ho L.L., Hsu W.M., Ku H.H. (2006). The immediate early 2 protein of human cytomegalovirus (HCMV) mediates the apoptotic control in HCMV retinitis through up-regulation of the cellular FLICE-inhibitory protein expression. J. Immunol..

[109] McCormick A.L., Roback L., Livingston-Rosanoff D., St Clair C. (2010). The human cytomegalovirus *UL36* gene controls caspase-dependent and -independent cell death programs activated by infection of monocytes differentiating to macrophages. J. Virol..

[110] Chien H., Dix R.D. (2012). Evidence for multiple cell death pathways during development of experimental cytomegalovirus retinitis in mice with retrovirus-induced immunosuppression: Apoptosis, necroptosis, and pyroptosis. J. Virol..

[111] Feldstein A.E., Gores G.J. (2005). Apoptosis in alcoholic and nonalcoholic steatohepatitis. Front. Biosci. J. Virtual Libr..

[112] Li H.P., Yuan C.L., Zho Y.C. (2015). Human cytomegalovirus inhibits apoptosis involving upregulation of the antiapoptotic protein Bag-1. J. Med. Virol..

[113] Chiou S.H., Liu J.H., Chen S.S., Liu W.T., Lin J.C., Wong W.W., Tseng W.S., Chou C.K., Liu C.Y., Ho L.L. (2002). Apoptosis of human retina and retinal pigment cells induced by human cytomegalovirus infection. Ophthalmic Res..

[114] Dou J., Li X., Cai Y., Chen H., Zhu S., Wang Q., Zou X., Mei Y., Yang Q., Li W. (2010). Human cytomegalovirus induces caspase-dependent apoptosis of megakaryocytic CHRF-288-11 cells by activating the JNK pathway. Int. J. Hematol..

[115] Shen Y.H., Utama B., Wang J., Raveendran M., Senthil D., Waldman W.J., Belcher J.D., Vercellotti G., Martin D., Mitchelle B.M. (2004). Human cytomegalovirus causes endothelial injury through the ataxia telangiectasia mutant and p53 DNA damage signaling pathways. Circ. Res..

[116] Terhune S., Torigoi E., Moorman N., Silva M., Qian Z., Shenk T., Yu D. (2007). Human cytomegalovirus UL38 protein blocks apoptosis. J. Virol..

[117] Qi R., Hua-Song Z., Xiao-Feng Z. (2013). Leflunomide inhibits the apoptosis of human embryonic lung fibroblasts infected by human cytomegalovirus. Eur. J. Med. Res..

[118] Biolatti M., Blangetti M., D’Arrigo G., Spyrakis F., Cappello P., Albano C., Ravanini P., Landolfo S., De Andrea M., Prandi C. (2020). Strigolactone Analogs Are Promising Antiviral Agents for the Treatment of Human Cytomegalovirus Infection. Microorganisms.

[119] Mo J., Zhang M., Marshall B., Smith S., Covar J., Atherton S. (2014). Interplay of autophagy and apoptosis during murine cytomegalovirus infection of RPE cells. Mol. Vis..

